# Prediction of winter wheat leaf chlorophyll content based on VIS/NIR spectroscopy using ANN and PLSR

**DOI:** 10.1002/fsn3.3071

**Published:** 2022-10-06

**Authors:** Vali Rasooli Sharabiani, Araz Soltani Nazarloo, Ebrahim Taghinezhad, Ibham Veza, Antoni Szumny, Adam Figiel

**Affiliations:** ^1^ Department of Biosystem Engineering, Fac. of Agriculture and Nat. Res. University of Mohaghegh Ardabili Ardabil Iran; ^2^ Moghan College of Agriculture and Natural Resources University of Mohaghegh Ardabili Ardabil Iran; ^3^ Department of Chemistry Wroclaw University of Environmental and Life Science Wrocław Poland; ^4^ Department of Mechanical Engineering Universiti Teknologi PETRONAS Perak Darul Ridzuan Malaysia; ^5^ Institute of Agricultural Engineering Wroclaw University of Environmental and Life Sciences Wrocław Poland

**Keywords:** artificial neural networks, leaf chlorophyll content, partial least squares, visible and near infrared, winter wheat

## Abstract

Visible–near‐infrared spectroscopy is known for its rapid and nondestructive characteristics designed to predict leaf chlorophyll content (LCC) of winter wheat. It is believed that the nonlinear technique is preferable to the linear method. The canopy reflectance was applied to generate the LCC prediction model. To accomplish such an objective, artificial neural networks (ANN), along with partial least squares regression (PLSR), nonlinear, and linear evaluation methods have been employed and evaluated to predict wheat LCC. The wheat leaves reflectance spectra were initially preprocessed using Savitzky–Golay smoothing, differentiation (first derivative), SNV (Standard Normal Variate), MSC (Multiplicative Scatter Correction), and their combinations. Afterward, a model for LCC using the reflectance spectra was developed by means of the PLS and ANN. The vis/NIR spectroscopy samples at the 350–1400 nm wavelength were preprocessed using S. Golay smoothing, D_1_, SNV, and MSC. The preprocessing with SNV‐S.G, followed by PLS and ANN modeling, was able to achieve the most accurate prediction, with the correlation coefficient of 0.92 and 0.97, along with the root mean square error of 0.9131 and 0.7305 receptivity. The experimental findings also revealed that the suggested method utilizing the PLS and ANN model with SNV‐S. G preprocessing was practically feasible to estimate the chlorophyll content of a particular winter wheat leaf area according to the visible and near‐infrared spectroscopy sensors, achieving improved precision and accuracy. The nonlinear technique was proposed as a more refined technique for LCC estimating.

## Graphical abstract

In this research, the amount of winter wheat leaf chlorophyll was nonlinearly predicted by visible–near‐infrared spectroscopy by methods of artificial neural networks (ANN) along with partial least squares regression (PLSR). The consequences of estimation of LCC for ANN model (𝑅^2^ = .97) were better than the PLSR model (𝑅^2^ = .92).

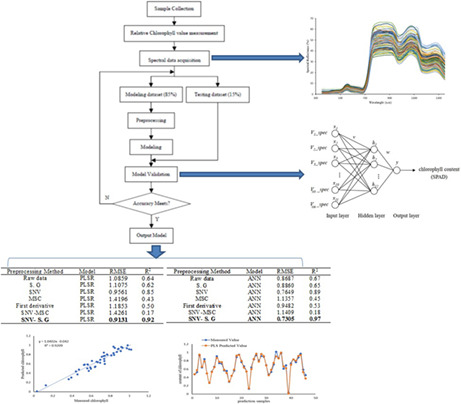

## 1 INTRODUCTION

The increasing population and changes in the annual yield of agricultural products have made food security an important international issue (Fuss et al., 2015). Opportune and precise estimates of the yield of agricultural products are essential for all aspects of human life (Alganci et al., 2014; Fang et al., 2011). Yield, along with protein, are the two most important factors in producing wheat. The concentration of protein is believed to influence wheat product quality. It is generally recognized that the genetic make‐up as well as environmental factors, such as N supply, water, and temperature, influence the amount of protein in wheat (Daniel & Triboı, 2002; Li et al., 2003; McDonald, 1992; Wang et al., 2014). Hence, a real‐time monitoring of plant N status, as well as wheat preharvest estimation for grain and protein yield or both, was able to help farmers in enhancing the N management approaches and producing yield or quality maps, or both, for successful marketing. Wheat yield prediction is essential in creating yield plots (Lukina et al., 2001; Mahey et al., 1991; Pinter et al., 1981; Raun et al., 2001). A common method for prediction uses the statistical analysis of data. Waste of time and user errors are negative features of this method. Therefore, a quick and precise method for prediction is required. As reported by Filella et al. (1995), remote sensing was able to offer affordable predictions of the N status in wheat plants for a large region. Visible and near‐infrared spectroscopy is an appropriate and rapid technique for calculating various crop factors (Chen et al., 2010; Fazeli Burestan et al., 2021; Haboudane et al., 2004; Mamo et al., 2003; Nigam et al., 2014; Thomas et al., 2008). The analysis of spectral data is performed under entire wave bands and multiple wavelengths quantitatively and qualitatively using the NIR spectroscopy method. Most chemometric multivariate statistical methods such as partial least squares regression (PLSR), principal component analysis, and regression (PCA and PCR) are the most frequently used techniques for NIR spectroscopy analysis (Hong, Chen, et al., 2019; Hong, Liu, et al., 2019; Walsh et al., 2004) and multiple linear regression (MLR). It should be noted that most multivariate statistical methods can solve the nonlinear trends (Wentzell & Montoto, 2003) specifically for the calibration of the nonlinear dataset (Despagne et al., 2000; Walczak & Massart, 1996). Hence, rather than utilizing such techniques, an artificial neural network or ANN was employed to calibrate the nonlinear dataset (Blanco et al., 1999; Flores et al., 2009; Franco et al., 2006; Makino et al., 2010; Marini, 2009; Micklander et al., 2006; Shao, Cao, & He, 2007; Shao, He, & Bao, 2007). ANN is an attractive technique in biological sciences owing to its prediction quality as well as its straightforwardness as opposed to process‐based models (Alvarez, 2009; Jørgensen & Bendoricchio, 2001). Calibration and classification in ANN have numerous advantages compared to traditional statistical techniques, particularly the ability for no‐predefined relations predictions (nonlinear effects and/or interactions). It is worth noting that ANN is extensively utilized and effectively implemented in the food business, for example, for the classification of cereal grain by means of morphological features (Dubey et al., 2006; Paliwal et al., 2001; Visen et al., 2002), bakery products thermal conductivity (Sablani et al., 2002), prediction of wheat flour Zeleny sedimentation volume (Razmi‐Rad et al., 2008), edible oils color determination (Kılıç, Onal‐Ulusoy, & Boyacı, 2007; Kılıç, Onal‐Ulusoy, Yıldırım, & Boyacı, 2007), and prediction of corn moisture, protein, as well as starch content (Fang et al., 2011). Many works have confirmed the capability of applying the vis/NIR spectroscopy technique to predict the quality of different agricultural products (Guo et al., 2020; Li et al., 2020; Towett et al., 2013; Zhong et al., 2021; Zhu et al., 2018). A spectrophotometer of N concentration of tomato leaves relies on the vis/NIR spectroscopy (Ulissi et al., 2011). The use of the spectroscopy technique for chlorophyll content calculation has been widely considered (Alganci et al., 2014; Jiménez‐Jiménez et al., 2012; Peng et al., 2014; Ulissi et al., 2011; Yao et al., 2013). Holer et al. (1983) considered the connection between the amount of the spectrum and crop nutrition. Fang et al. (2007) suggested a method to estimate relative leaf nutrition. They proved that such a method could estimate the relative content of leaf chlorophyll using spectral assessment. Jamshidi et al. (2012) applied MSC as well as SNV to spectral dataset processing, along with the PLS modeling approach for the nondestructive prediction of Valencia‐oranges taste qualities according to the near‐infrared and visible spectroscopy. Visible and infrared sensor of near image was used by Zhang et al. (2015) to predict the content of soluble protein from the leaves of oilseed rape. GAPLS or Genetic algorithm–partial least square was utilized to select the sensitive wavelength. Yao et al. (2013) examined the correlation between spectral data and the content of rice chlorophyll by comparing the impacts of modeling approaches from PCR, PLS, SMLR, and BPNN. They demonstrated that the PLS model of rice leaf pigment using near‐infrared spectroscopy was able to improve its performance. Since PLS and BPNN algorithms performed important roles in the modeling application for agricultural products spectral analysis, this study used PLS and BPNN algorithms to examine the correlations between the winter wheat leaf spectral data and the relative chlorophyll content. Hansen et al. (2002) attempted to employ early, recurring remote sensing multispectral dataset to estimate grain production as well as quality of winter wheat and spring barley. The yield estimation was found to be decent, yet the protein content estimation was relatively poor. Currently, nondestructive, fast, sensitive, straightforward, chemical‐free analytical approaches are favored for numerous analytical applications. Evaluating the NIR spectrum by means of chemometric methods combined with artificial neural networks has a great potential to meet such a requirement. The present study aims to examine the ANN ability to estimate protein content as a quality parameter as well as winter wheat yield employing the sample NIR spectrum. For every parameter, a single network was developed and trained. Also, the optimal network parameters were established for high‐level accuracy parameter forecast. The capability of network prediction was assessed by evaluating the measured data with the estimated ones.

## 2 METHODOLOGY

### 2.1 Study site

The experiments for the winter wheat field were performed in the farming area of the Ardabil city, Iran. A traditional winter wheat type “Sabalan” was grown on the field for three uninterrupted years. The total area of the field was 40 × 120 m^2^ and it was split into eight blocks. Reflectance data were acquired by employing a SpectroradiometerFieldSpec3 (FS3) (Analytical Spectral Devices, Inc.).

### 2.2 Spectroradiometer

**FIGURE 1:**
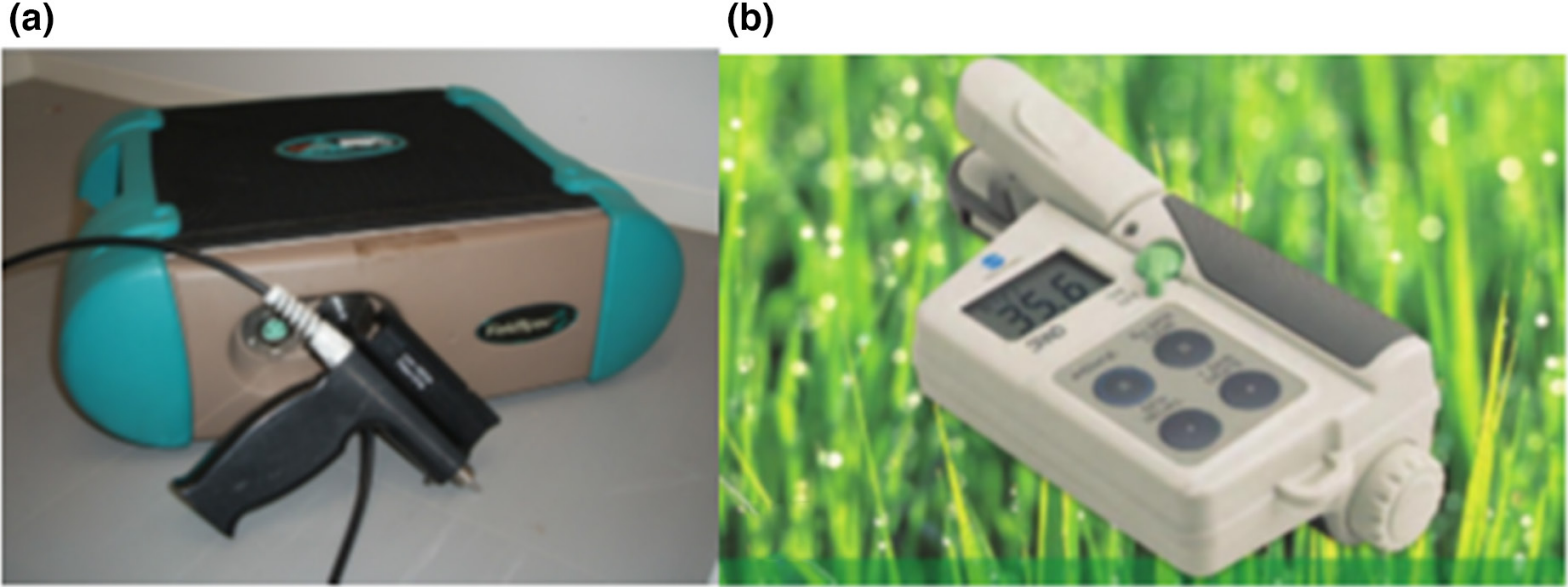
(a) Spectroradiometer 3 (FS3) (b) SPAD meter for SPAD value measurement

In the present study, a spectroradiometer FS3 with a high resolution was utilized to acquire the spectral dataset. FS3 is a hyperspectral sensor created for solar dataset collection (reflectance, radiance, and irradiance). It can measure the spectral reflectivity from 350 to 2500 nm, having 1.4 and 2 nm sampling intervals in the spans of 350–1050 nm and 1000–2500 nm, respectively, thus having a total of 2150 wavelengths. The wireless connection facilitates the remote control to collect data up to 50 m. Moreover, a laptop was used to obtain data for the FS3 control using special software. Also, a 1.5 m fiber cable measured from different angles has been equipped with a 25° standard viewing angle. The mounting lens could be adjusted to 1°, 3°, 8°, to 10°. In the present study, 25 degrees was selected as the viewing angle. When the sensor's height was 150 cm higher than the ground, the detection area was around 0.4 m^2^. Furthermore, a solar sensor was employed to provide the background light reference data. The calibration utilizing a typical whiteboard was performed just prior to the measurement of the reflectance value. Figure 1a illustrates the FS3 (Su, 2013), while SPAD‐502 chlorophyll meter is presented in Figure 1b.

### 2.3 SPAD value measurements

The chlorophyll content found in the leaf is a crucial factor for both agriculturists and plant physiologists. It is a sign of leaf senescence and plants’ nitrogen status. The chlorophyll content can be changed to adapt to environmental conditions. Several nondestructive techniques can determine the leaf's chlorophyll content, such as measuring the leaf transmittance (T) at a specific suitable wavelength(s). Several devices using this principle are offered today on the market, such as a SPAD‐502 chlorophyll meter, as shown in Figure 1b. This chlorophyll meter gauges light intensity that is transmitted via the leaf sample at two different wavelengths, i.e., 650 and 940 nm, employing LED with an estimated half‐width of the emission spectrum of 15 and 50 nm, respectively. The display will show an M value relative to the SPAD units (Naus et al. 2010).

The M quantity can be calculated using the following equation (Equation 3.1):

Here, I (650) and I (940) are defined as signals with no sample, I′ (650) and I′ (940) signals with the sample, and log are common logarithms. The quantity k, known as the coefficient of confidential proportionality, is the relative SPAD units. C is nothing but the compensation value and can be adjusted in the software. For practicality, the transmittance T negative common logarithm at 650 nm associated with that at 940 nm is linearly proportional to the chlorophyll content. In every single experiment, the SPAD‐502 chlorophyll meter record must be calibrated for actual leaf chlorophyll content since their correlation can be different between species or cultivars and among plants cultivated under different environmental conditions. Furthermore, the manufacturer states that SPAD readings can differ depending on the type of the leaf. The SPAD value was measured using a SPAD meter. The SPAD meter determined the relative quantity of chlorophyll existence by gauging the leaf absorbance in two regions of wavelength (red and near‐infrared). Therefore, it can indicate the presence of chlorophyll content in plant leaves. Referring to SPAD 502 catalog, there is a high correlation (*R* ^2^ > .9) between the SPAD value and the concentration of leaf nitrogen; thus, it has been extensively employed to detect crop chlorophyll and nitrogen content. It is also used as guidance for plant health as well as top dressing (Zhang et al., 2003).

## 3 DATASET ARRANGEMENT

**FIGURE 2:**
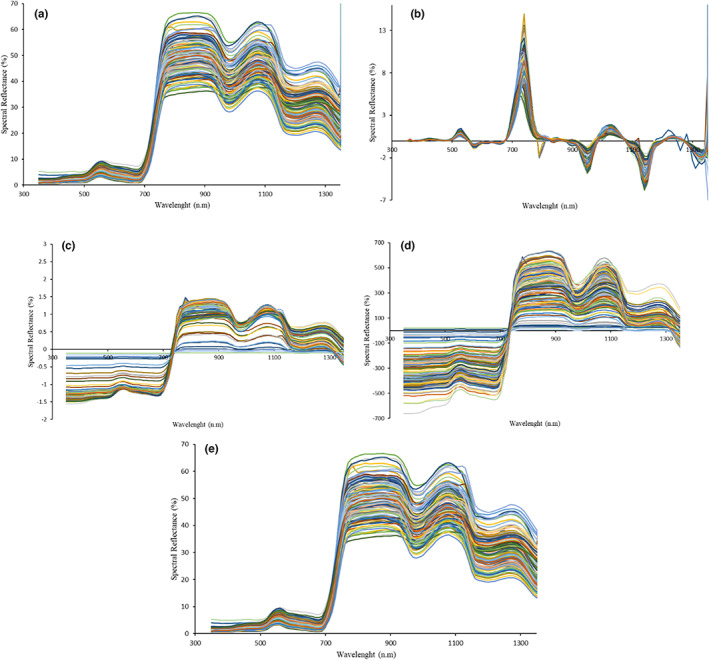
Reflectance spectra with different preprocessing including of: the Savitzky–Golay smoothing; (a), the first derivative; (b), standard Normal variate; (c) and multiplicative scatter correction; (d), raw samples (e)

The wheat leaves are chosen from various regions nearby Ardabil town in Ardabil Province (Iran). A total of 160 sample leaves, 80 prior to fertilization and 80 samples following fertilization, were selected. The sample diversity, which encompasses various arid and semiarid areas along with different affecting factors for the wheat growth, can prevent the issue of single farmland environments and avoid single sample modeling. All the samples were gathered from June 1 to 15 2015 and 2016 (80 samples for each year), which is the duration of wheat jointing. It should be noted that wavelength reflectance spectral data ranging from 350 to 1350 nm were chosen to analyze the study and model. The samples' raw reflectance spectra are displayed in Figure 2e. The spectral wavelength is the abscissa, while the spectral reflection coefficient is the vertical axis. The peak of reflection around 550 nm is the reflection region of green light. Moreover, the red edge region is a band with the length of 690–720 nm, showing a negative relationship between the content of chlorophyll and its peak sizes (Horler et al. 1983).

### 3.1 Statistical analysis

#### 3.1.1 Reflectance data preprocessing

A substantial amount of spectral data is normally acquired from spectral devices and produce valuable analytical insight (Blanco et al., 1999). Yet, the spectrometer data encompass background noise and information apart from the sample data. To acquire dependable, accurate level, and stable calibration models, performing spectral data preprocessing prior to modeling using PLS is essential. Spectral preprocessing methods are needed to eliminate any unrelated data. Numerous preprocessing methods for spectral data were established in recent times.

There are several spectrum preprocessing methods for spectral analysis to eliminate unwanted variations, which may negatively affect the interpretation of a model. SNV (Standard Normal Variate) and MSC (Multiplicative Scatter Correction) are some of the most commonly utilized preprocessing techniques for spectral data. One advantage of these techniques is that they do not need a response variable in the preprocessing stage, which is normally required in another method, such as orthogonal signal correction (OSC). SNV and MSC take away both additive as well as multiplicative baseline variation without changing the spectra shape. In addition to SNV and MSC, Savitzky–Golay smoothing and differentiation (first derivative) were also used in this study. Therefore, in the present analysis, original spectral data having four kinds of preprocessing techniques were utilized, including Savitzky–Golay smoothing (Figure 2a), differentiation (first derivative) (Figure 2b), SNV (Standard Normal Variate) (Figure 2c), and MSC (Multiplicative Scatter Correction) (Figure 2d), and samples of raw reflectance spectra (Figure 2e).

#### 3.1.2 Regression analysis

Partial least squares regression (PLSR) applied in Unscrambler version 10.2 was utilized to establish calibrations between reflectance spectra and crop variables. PLSR is a popular method in remote sensing, chemometrics, and spectral data processing for overcoming a large amount of data comprising very well‐correlated variables (e.g., Nanni & Dematte, 2006; Vasques et al., 2009). It is a full‐spectrum technique using information from the entire wavelengths in the original spectrum to establish a calibration algorithm. The calibration of PLS generates a set of new variables known as factors, which are uncorrelated and describe the variation in response and predictor variables (Beebe & Kowalski, 1987). A crucial stage in PLS regression is determining the optimum number of factors that represent the calibration data to the highest degree with no overfitting. This involves a validation stage normally performed either via a leave out one (LOO) cross‐validation technique or splitting the dataset into independent calibration and validation sets. Hence, in the present study, several independent combinations and validation datasets were applied before we selected the best combination model, which has the highest determination coefficient (*R* ^2^), and the lowest root mean square error (RMSE). The use of Unscrambler software was able to determine a recommended number of factors to reduce the prediction error. The final calibration model was acquired from the full data employing these factors' numbers. Following the preprocessing of the spectral data, the 160 samples were divided into two different groups. As many as 40 and 120 samples were arbitrarily selected as the prediction and calibration sets. The quantitative analysis model between the values of chlorophyll and the spectral dataset was developed in the band length ranging from 350 to1350 nm on PLS. Finally, the prediction dataset of the SPAD values was then predicted.

#### 3.1.3 Artificial neural networks (ANN) model

**FIGURE 3:**
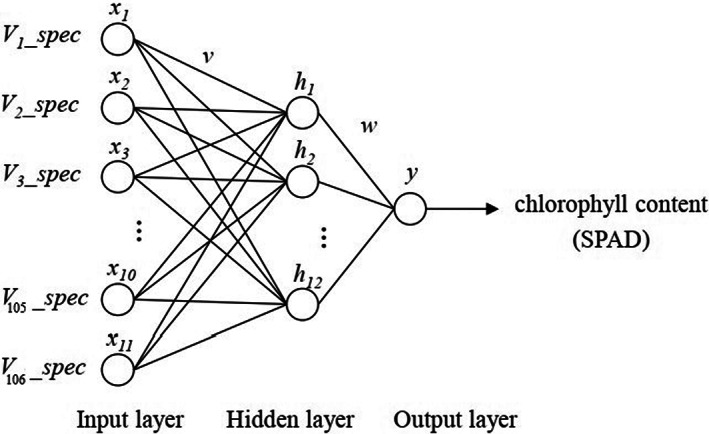
The ANN structure for calculating wheat quality parameters

Artificial neural networks is a statistical learning method inspired by humans neurologically. ANN has a robust capability for pattern recognition, thus allowing it to learn and re‐learn solving a complicated system having multivariable inputs and outputs. BPNN is a well‐known type of neural network. It has the benefits of nonlinearity, fault tolerance, parallel processing, self‐adaptation, as well as self‐learning. Hence, the BPNN is considered superior for various applications, such as prediction, classification, data fitting, and system modeling. A standard BPNN has one input, hidden, and output layer. The input layer is nothing but a layer that is linked to the external system. In contrast, the output layer is connected to the external system with the output neurons' number directly correlated to the task type. It should be noted that the ANN training should be established. The hidden layer consists of a group of neurons with an activation function. It is an intermediate layer connecting the input and output layers. The algorithm of BPNN is developed to reduce the root mean square error of the desired output. It is important to remember that the number of nodes in the hidden layer greatly impacts the BPNN performance. If the neurons' number is smaller than the work complexity, the ANN model cannot fully represent the correlations between the input and output variables. If a bigger than necessary number of neurons are selected, the issue of overfitting may arise. Overall, the development of trained ANN involves the data generation for training/testing steps, the development of the architecture, the ANN training/testing, and the assessment of the ANN outcomes, resulting in the selection of an optimally trained ANN model. The ANN architecture is illustrated in Figure 3. The data were mainly produced by measuring the absorbance values for 160 wheat total samples with the NIR spectroscopy ranging from 350 to 1400 nm for an interval of 10 nm. As many as 120 of the 160 data units were utilized as training data, while the remaining 160 units were selected as the testing data to develop an ANN model using MATLAB R2017. The calibration models were carried out by means of a generalized feed‐forward and multilayer perceptron network. Transfer function, neuron's number from one to 10 in the hidden layer, and epoch number from 100 to 10.000) were examined to reduce the mean square error (MSE) between outputs and targets. The optimum network with the lowest value of MSE and the highest *R* ^2^ between measured and predicted data was chosen for further purposes.

### 3.2 Data analysis

#### 3.2.1 Partial least squares regression model (PLSR)

To avoid errors from magnitudes variables differences, each variable was normalized between 0 and 1 before the PLSR was performed (Chen et al., 2013). A leave‐one‐out cross‐validation strategy was employed for an appropriate number of principal components selection and to avoid overfitting. While designing the PLSR model, the utilized principal component was altered from 1 to 14. The principal components number with the minimum value of RMSE and the maximum *R* ^2^ were chosen as the best PLSR model. The model of final PLSR was established by employing the chosen principal components along with the entire samples of the calibration database.

#### 3.2.2 ANN model

In the present study, a back‐propagation artificial neural network with three layers was utilized, i.e., input, hidden, and output. This network works better compared to other networks (Farifteh et al., 2007; Hornik et al., 1989). Like the PLSR model, each reflectance variable was normalized between 0 and 1. Generally, a principal component analysis (PCA) is employed to compute and determine the optimized variables of the reflectance bands' principal component to decrease the number of variables. The input nodes in the input layer incorporate the variables of principal component vectors in the role of the input data, matching the one output node with vectors of the wheat chlorophyll content in the function of the target data in the output layer. The principal components number and hidden nodes as well as the kinds of the algorithm to convert from input to hidden nodes and from hidden to output nodes were established by employing a leave‐one‐out cross‐validation technique. Throughout the procedure, the number of principal components shifted from one to 10 since 10 principal components can signify 99.9% of variables in the matrix of an independent variable. In addition to the principal component, the number of hidden nodes was fixed between 1 and 5 as excessive hidden nodes can result in overfitting issues (Huang & Foo, 2002). Furthermore, three standard formulas were chosen for the transformation from input to hidden nodes as well as from hidden to output nodes. They were the tan‐sigmoid, log‐sigmoid, and linear function. The last ANN model was created utilizing the chosen number of principal components, the number of hidden nodes, and the transformation algorithm from the input to the hidden nodes. It should be noted that the transformation algorithm from hidden to output nodes used the entire samples from the calibration database. The determination coefficient and RMSE between the estimated and actual chlorophyll were then calculated. A code to carry out the above process was written in MATLAB 2017.

## 4 RESULTS AND DISCUSSION

### 4.1 PLSR model

Given the cross‐validation outcomes of the PLSR based on the number of the principal components, the lowest value of RMSE and maximum *R* ^2^ were achieved using eight principal components (Table 1). Hence, eight principal components were employed for the final PLSR model design.

**TABLE 1:** Cross‐validation outcomes of the PLSR model with various principal components number

Number of PCs	RMSE	*R* ^2^
1	1.2198	.26
2	1.167	.39
3	1.1958	.37
4	1.2978	.26
5	1.3242	.31
6	1.3134	.94
7	1.1688	.72
8	0.9402	.92
9	1.107	.72
10	1.1394	.87
11	1.3068	.70
12	1.3284	.70
13	1.3362	.68
14	1.3368	.68

Table 2 shows the dataset model's predictive capabilities. It should be noted that the predictive performances of SNV‐S. G and SNV are better compared to MSC. The best outcome, the *R* ^2^ with .92 and the RMSE with 0.9131, has been obtained by SNV‐S. G.

**TABLE 2:** Testing dataset predictive capabilities using various approaches and modeling with the PLS

Preprocessing method	Model	RMSE	*R* ^2^
Raw data	PLSR	1.0859	.64
S. G	PLSR	1.1075	.62
SNV	PLSR	0.9561	.85
MSC	PLSR	1.4196	.43
First derivative	PLSR	1.1853	.50
SNV‐MSC	PLSR	1.4261	.17
SNV‐S. G	PLSR	0.9131	.92

**FIGURE 4:**
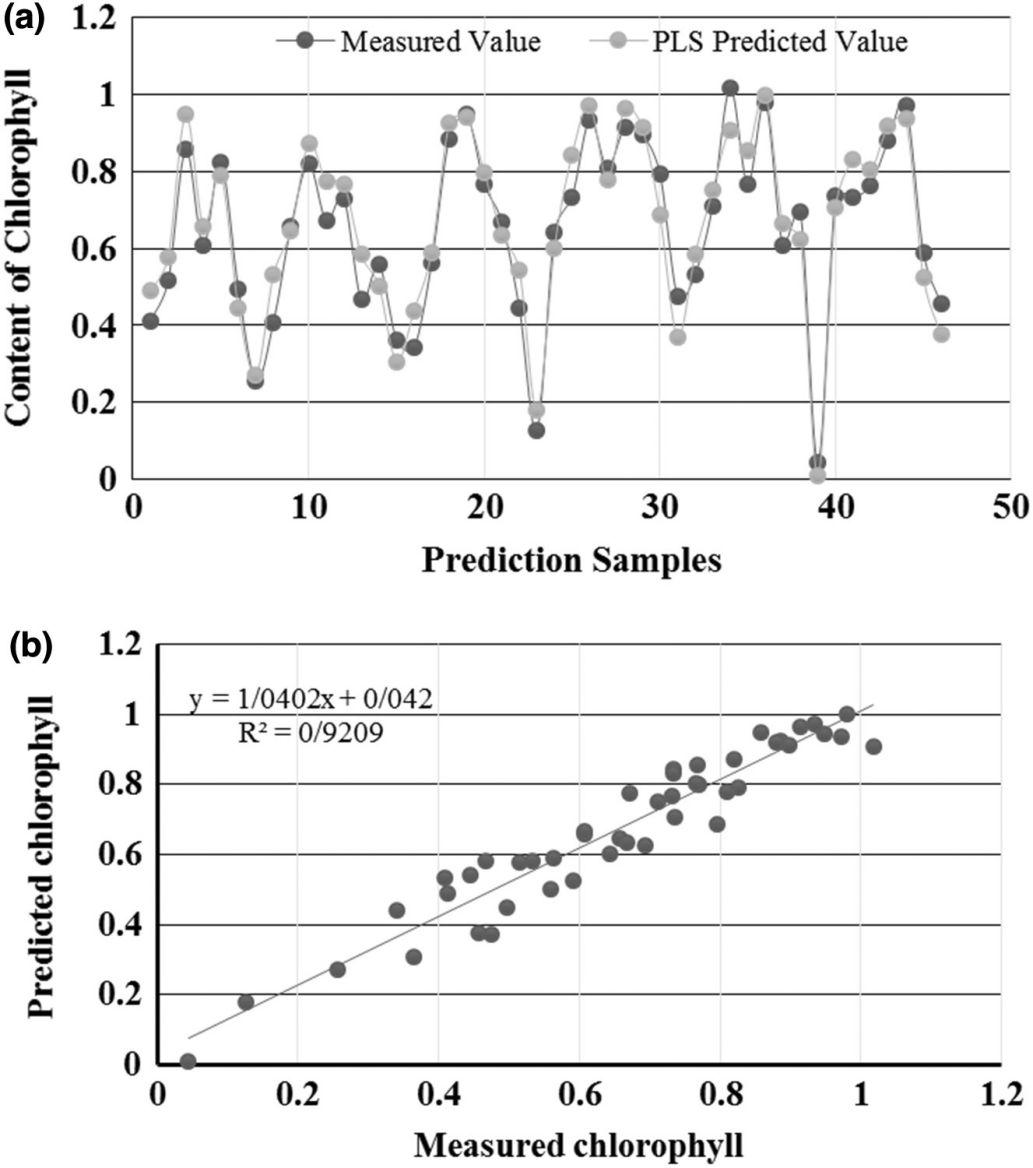
Actual (measured) and predicted values of the 46 testing samples using PLS (a), predicted vs measured chlorophyll using PLSR (b)

Following the processing of sample spectral data by SNV‐ S. G, the PLS predictive model was employed for the chlorophyll content prediction of every 46 samples. The actual (measured) and predicted values of 46 datasets are displayed in Figure 4a. The horizontal axis is the prediction samples, whereas the perpendicular axis is the chlorophyll content, with *R* ^2^ .92 and the RMSE 0.9131.

According to the SNV‐S. G preprocessing technique, the outcomes of the PLSR can be seen in Figure 4b. It is shown that the model of PLSR provides decent outcomes, having the value of RMSE of 0.9131 with an *R* ^2^ of .92.

### 4.2 ANN model

The artificial neural networks model's minimum value of RMSE and the maximum *R* ^2^ were achieved by combining eight principal components and two hidden layers. A linear function was employed as the transformation algorithm for the input‐hidden layer, while the function of log‐sigmoid was utilized for the hidden‐output layer. Given the large amount of data, the outcome combinations were not provided here. Based on the database calibration, the model of the artificial neural network was created by means of the above‐chosen number of principal components, hidden node, and algorithm of transformation for input‐hidden nodes as well as for the hidden‐output node. Table 3 shows the model predictive capabilities for the datasets. It is shown that the predictive performances of SNV‐S. G and SNV provide better results as opposed to those of MSC. The best outcome, the *R* ^2^ of .97 and the RMSE of .7305, was achieved by SNV‐ S. G.

**TABLE 3:** Testing dataset predictive capabilities using ANN various methods and modeling

Preprocessing method	Model	RMSE	*R* ^2^
Raw data	ANN	0.8687	.67
S. G	ANN	0.8860	.65
SNV	ANN	0.7649	.89
MSC	ANN	1.1357	.45
First derivative	ANN	0.9482	.53
SNV‐MSC	ANN	1.1409	.18
SNV‐S. G	ANN	0.7305	.97

**FIGURE 5:**
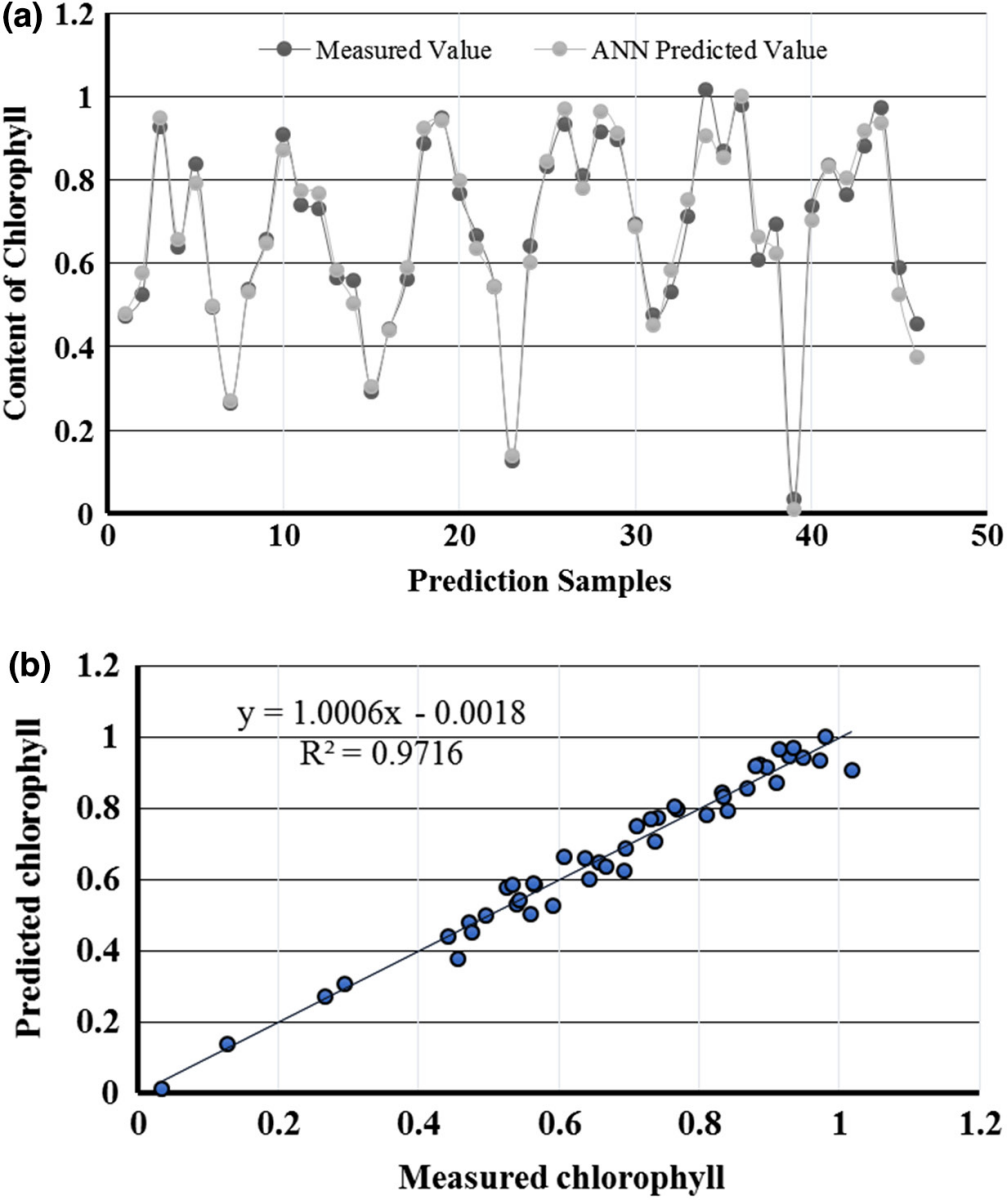
Measured and predicted chlorophyll value of ANN model (a), measured and predicted values of ANN testing dataset (b)

Following the processing of sample spectral data by SNV‐S. G, the predictive ANN model was employed for chlorophyll content prediction for every 46 samples. The measured (actual) and predicted values of 46 samples are given in Figure 5a. The horizontal axis are the prediction samples, while the vertical axis is the chlorophyll content. The *R* ^2^ value of the model is .97 with an RMSE of 0.7305.

From the SNV‐S. G preprocessing approach, the outcomes of the ANN model are shown in Figure 5b. For such a model, the PLSR showed promising results, with the value of RMSE of 0.7305 and *R* ^2^ of .97.

Evaluating the models of PLSR and ANN, the ANN model showed improved results, providing a higher *R* ^2^ and lower RMSE value. Since the transformation function was not linear, the model of ANN was assigned as a nonlinear function. In the meantime, the model of PLSR was a linear function. Therefore, the correlation between the reflectance of the wheat canopy with its chlorophyll was well described by a nonlinear equation.

## 5 CONCLUSION

The analysis reveals that the nonlinear technique is more appropriate for establishing LCC estimation models along with directly utilizing the canopy reflectance dataset. By means of a high‐resolution spectroradiometer, FieldSpec3 (FS3) wheat LCC data, PLSR, and ANN were carefully chosen to characterize a linear and nonlinear approach. They were evaluated for the winter wheat LCC predictions. The findings revealed that ANN showed a superior estimation for wheat LCC, indicated by a maximum value of *R* ^2^ as well as minimum RMSE values. These outcomes offer valuable knowledge for the design of appropriate numerical LCC estimation models. Evaluating the predictive efficiency established on the PLS and ANN model, along with the preprocessing by means of MSC, SNV, SWS‐MSC, and SWS‐SNV, it is worth noting that the SWS‐MSC or SWS‐SNV is better as opposed to merely employing MSC/SNV, irrespective of the diverse parts of the sample data or model. Both the measured and predicted findings showed that combining multiple preprocessing for spectral data is an effective technique to enhance accuracy. The preprocessing with SNV‐S. G followed by PLS and ANN modeling could accomplish the most accurate prediction with the correlation coefficient of 0.92 and 0.97, along with the root mean square error of 0.9131 and 0.7305 receptivity. The experimental findings also revealed that the suggested method utilizing the PLS and ANN model with SNV‐S.G preprocessing was practically feasible to estimate the chlorophyll content of a particular area of winter wheat leaf according to the visible and near‐infrared spectroscopy sensors, achieving an improved precision and accuracy. The suggested method may be employed for an available hand‐held chlorophyll device or an on‐line field application for future study.
